# Essentials of Dermatology

**Published:** 1991-06

**Authors:** M.G. Wilson


### ESSENTIALS OF DERMATOLOGY, Third edition
**J.L. Burton,**
Churchill Livingstone, Price £10.00

The second edition of this book was well received by the reviewer in our predecessor the Bristol Medico Chirurgical Journal (April 1986, p. 44). The third edition has been updated and enlarged from 260 to 298 pages and the number of colour photos increased from 40 to 50. The author now regards it as suitable for M.R.C.P. candidates and for general practitioners as well as for undergraduates. Thanks to subsidy from a drug firm the cost has remained relatively unchanged and at £10 remains the cheapest book in this field. With its easy readability', and its touches of humour', it can still in the words of our previous reviewer be strongly recommended'.

M.G. Wilson

